# Some Questions to Our Chinese Colleagues Pioneering Research Into Coronavirus Disease 2019 (COVID-19)

**DOI:** 10.3389/fmed.2020.594623

**Published:** 2020-12-08

**Authors:** Claudia Stöllberger, Maria Winkler-Dworak

**Affiliations:** ^1^Medizinische Abteilung, Klinik Landstrasse, Vienna, Austria; ^2^Wittgenstein Centre for Demography and Global Human Capital, Vienna Institute of Demography of the Austrian Academy of Sciences, Vienna, Austria

**Keywords:** statistics, infection, electrocardiogram, mortality, cardiology

## Abstract

A pandemic has developed, so physicians worldwide are particularly interested in the experiences of their Chinese Colleagues which are frequently cited. To assess the long-term pulmonary, cardiac, neurologic, and psychiatric consequences after COVID-19, the outcome of patients included in the early publications and the association with baseline findings is of particular interest. Thus, we review the methods of early Coronavirus disease 2019 (COVID-19) publications. Reports published before March 19th 2020, comprising >40 patients were included, considering especially cardiologic aspects. It remains unclear whether patients were described several times, or they were different patients. Only patients with confirmed COVID-19 were described, and no differences in findings of patients with initially suspected and later confirmed, or excluded infection. It remains unclear in how many cases information was missing, since missing values were not reported. Medication before hospital admission, level of education and occupation, household size and composition, weight or body mass index are lacking. No details about electrocardiographic findings are given. Patients still in follow-up, constituting the major part of observations, were excluded. The data should be re-analyzed. A comparison between confirmed and excluded cases could be carried out. By now, in November 2020, the reported patients will most probably have recovered. Thus, it would be possible to differentiate prognostic indicators more precisely. Laboratory tests and electrocardiograms could be analyzed in more detail to shed light on the spectrum of this disease and to solve some of the unanswered questions related with COVID-19.

## Background

At the beginning of 2020, symptoms, laboratory findings, complications, and outcome of patients with Coronavirus disease 2019 (COVID-19) were reported from China. Since then, a pandemic has developed, and accordingly, a great number of articles have been published. On November 18th 2020, the search term “COVID-19” yielded 75,182 results in PubMed. Since the first COVID-19 infections were reported from Wuhan in China, physicians worldwide are particularly interested in the experiences of their colleagues and the early publications are frequently cited (Table 1) (1–6).

**Table 1:** Studies from China about COVID-19 published until March 19th, 2020.

**Authors**	**Hospital/Place**	**Sample size**	**Design**	**Statistical analysis**	**Methodological concerns**	**Missing information**	**PubMed citations on November 18^**th**^ 2020**
(1)	Jin Yin-tan Hospital (Wuhan, China)	41	Group comparisons between ICU (*n* = 13) and non ICU patients (*n* = 28) admitted to hospital between December 16, 2019 to Jan 2, 2020.	Mann Whitney *U*-test, chi2 test, Fisher's exact	Definition of comorbidities are missing, no reference values for laboratory tests.	Weight, BMI, ECG, CK levels, BNP levels, medication at admission, urine tests. N/A information is missing.	7,023
(2)	Jin Yin-tan and Tongji hospital (Wuhan, China)	150	Group comparison between discharged (*n* = 82) and deceased (*n* = 68) patients.	*t*-test, Mann Whitney–Wilcoxon test, chi2 test, Fisher's exact test	Definition of comorbidities are lacking. Recruitment of patients and time period of enrollment is unclear. No survival analysis. The term “myocarditis” is used without definition.	Weight, BMI, ECG, BNP levels, medication at admission, urine tests. N/A information is missing.	850
(3)	Jin Yin-tan hospital (Wuhan, China)	52	Group comparisons of deaths (*n* = 32) and survivors (*n* = 20) of critically ill adult patients with pneumonia admitted from December 24, 2019 to January 12, 2020; final date of follow-up February 9, 2020.	*t*-test, Wilcoxon rank sum test, chi2 test, Fisher's exact test, Kaplan–Meier curves	Definition of comorbidities are missing, no reference values for laboratory tests. Cardiac injury is only defined by troponin elevation.	Weight, BMI, ECG, BNP levels, medication at admission, urine tests. N/A information is missing.	1,850
(4)	Zhongnan (Wuhan, China)	138	Group comparisons of ICU (*n* = 36) and non ICU (*n* = 132) patients with pneumonia admitted from January 1 to January 28, 2020; final date of follow-up February 3, 2020.	*t*-test, Mann Whitney–Wilcoxon test, generalized linear mixed model, chi2 test, Fisher's exact test	Definition of comorbidities are missing. “Arrhythmia” is not defined.	Weight, BMI, ECG, BNP levels, medication at admission, urine tests. N/A information is missing.	4,179
(5)	552 hospitals including 132 patients from Jin Yin-tan Hospital (Wuhan, China)	1,099	Group comparison of non-severe (*n* = 926) and severe patients (*n* = 173) and of those experiencing a primary endpoint event (*n* = 67) and not (*n* = 1,032) out of 7,735 COVID-19 patients admitted from December 11, 2019 to January 29, 2020. Composite end points: ICU, mechanical ventilation or death. Patients censored with no outcome by January 31, 2020.	Descriptive, median and interquartile range, count, and percentage	Definition of comorbidities are missing, no reference values for laboratory tests.	Weight, BMI, ECG, medication at admission, urine tests, cardiac problems. N/A information is partially missing.	4,282
(6)	Jin Yin-tan Hospital and Wuhan Pulmonary Hospital (Wuhan, China)	191	Group comparisons between survivors (*n* = 137) and non-survivors (*n* = 54) out of 813 adult COVID-19 patients, admitted from December 29, 2019 to January 31, 2020, who died or were discharged by January 31, 2020.	Mann–Whitney *U*-test, χ^2^ test, or Fisher's exact, logistic regression	Definition of comorbidities are missing. Censored patients not included.	Weight, BMI, ECG, medication at admission, urine tests, cardiac problems. N/A information is missing.	4,112

*ICU, Intensive care unit; BMI, Body mass index; ECG, electrocardiogram; BNP, brain natriuretic pepetide; N/A, not available*.

Follow-up data about the consequences of COVID-19 infection on the health of affected patients are not yet available. There are indications for long-term pulmonary, cardiac, neurologic, and psychiatric illnesses and disabilities after COVID-19 (7). In that respect, again, the colleagues in Wuhan could be pioneers in reporting the long-term outcome of patients included in their early publications and relate them with clinical and instrumental findings at baseline. These baseline-data, however, were obtained under pressure of an evolving epidemic, which is also reflected by a high rate of published errata (1–3, 6). There are concerns about the methodological quality of the data (8).

Thus, the present article endeavors to review methods and results of early COVID-19 publications. We have included articles from Wuhan, China, published before March 19th 2020, which provided clinical details about >40 patients in English language (1–6). Not considered were case reports, epidemiologic, virologic, or radiologic studies without clinical data, publications with <40 patients and publications from which it was evident that they are duplicate publications. Since one of the authors is a cardiologist, the cardiological aspects were especially considered.

## Main Body

### Several Publications From the Same Hospital

Reviewing the articles, it became obvious that four of them originate from the same institution (1–3, 6). It remains unclear whether patients were described several times, or whether they were different patients.

### Suspected Covid-19 Cases

The articles described only patients with confirmed COVID-19. In clinical routine, however, it is important to differentiate between COVID-19 and other diseases. It would be very useful to know differences in symptoms and laboratory findings in patients with initially suspected and later confirmed, or excluded, COVID-19 infection.

This issue is mentioned in one article: “The absence of fever in COVID-19 is more frequent than in Severe Acute Respiratory Syndrome Coronavirus (SARS-CoV) and Middle East Respiratory Syndrome Coronavirus (MERS-CoV) infection, so afebrile patients may be missed if the surveillance case definition focuses on fever detection” (5). In a further article, the consequences of atypical presentations are described: “A total of 14 patients (10%) initially presented with diarrhea and nausea 1 to 2 days prior to development of fever and dyspnea…. One patient in the current study presented with abdominal symptoms and was admitted to the surgical department. More than 10 health care workers in this department were presumed to have been infected by this patient…” (4).

### Missing Values

The data in all included studies were collected retrospectively. It remains unclear, however, in how many cases information was missing, since just one publication reported missing values in some, not all, tables (5).

### Other Symptoms

Ageusia or anosmia were not reported in the included articles, but only in more recent ones from China (9) and Italy (10, 11). Why were these not reported in the included articles? One answer could be that symptoms were assessed in accordance with the International Severe Acute Respiratory and Emerging Infection Consortium (ISARIC) case record form, as indicated in the methods section of the articles. In this form, there is no space for “other symptoms” (12).

### Missing Baseline Information

Data about medication before hospital admission are lacking, thus no information can be obtained whether specific drugs render the patients more prone to a severe course of the disease. This issue, relating angiotensin-converting enzyme inhibitors, angiotensin II receptor blockers, and statins has been extensively discussed in the meantime (13, 14).

Data about level of education and occupation, household size and composition are not reported. Neither weight nor body mass index of the patients are reported. The reason might be the above-mentioned ISARIC case record form, as it does not require such information. Among the comorbidities, the terms “obesity” and “malnutrition” “as defined by the clinical staff” are registered in the case record form, but not the body mass index as an objective measure of obesity. Whether obesity influences the prognosis in COVID-19 is a still controversially assessed issue, and it is uncertain whether ethnic and socioeconomic differences might play a role for the discrepant findings (15–17).

We miss urine analysis data, which might help to characterize the type and pathomechanism of renal failure in COVID-19 (18). Is the frequently encountered hypalbuminemia due to renal loss of protein? Again, this lack of data may be related to the ISARIC case record form, where no such data are requested.

### Lack of Information About the Heart

“Cardiac injury” is frequently reported in COVID-19 patients. The definition for “cardiac injury” was adopted from a publication in which cardiac biomarkers, electrocardiography (ECG), or echocardiography among hospitalized patients infected with influenza A (H7N9) virus had been reported (19). “Myocarditis” is reported in one publication, however, it remains unclear how it was diagnosed (2). In another publication, arrhythmia is mentioned, but no description is given (4). In yet another publication, “cardiac injury” is only defined by elevations of the troponin (3).

No details about ECG findings are given in any of the included articles. Whereas, echocardiography should only be performed if it is expected to provide clinical benefit (and not as screening investigation), because of close proximity of patient and echocardiographer; this is not the case with ECG (20). An ECG is easy to perform and yields useful information about the cardiac condition, especially in patients with preexisting cardiovascular diseases. It would be of great interest to know how often and which types of arrhythmias were registered, the prevalence and dynamics of ST-elevations or depressions, and their association with cardiac biomarkers like troponin or natriuretic peptides. Up to now, our knowledge about ECG findings in COVID-19 patients from China is limited to small series comprising 63 (21), 102 (22), and 112 patients (23).

### Statistical Issues

The negligence of relevant variables can have important consequences. The variables may be confounders and the omission of these confounders will produce biased results. Moreover, the consequences might be enduring in future analyses, as model-building is often based (and even advised in some textbooks) on results of prior studies (24, 25). The selection of patients is unclear or biased (4, 5). In addition, patients still in follow-up, constituting the major part of observations, were excluded from the sample in two studies (3, 6). This phenomenon, that patients are still hospitalized when the article is written, is not only encountered in publications from China but also from Europe and the U.S.A (17, 26).

## Conclusions

It remains uncertain if such missing clinical information on COVID-19 patients was due to a rush to publish as rapid due to competition or due to underreporting. The retrospectively collected data, obtained under pressure of an evolving epidemic, will not have the quality of prospectively collected information. Nevertheless, it would be worthwhile to re-analyse and re-collect data of the patients included in these early studies. By doing this, a comparison of symptoms and findings between initially suspected and eventually confirmed and excluded COVID-19 cases could possibly be carried out. This would be helpful for physicians in emergency rooms worldwide. Moreover, by now, in November 2020, the reported patients will most probably have experienced an outcome, i.e., either recovered and are discharged from hospital or died from COVID-19. Thus, it would be possible differentiate indicators for survival or death more precisely with the complete observations in the full samples. Furthermore, laboratory tests, ECG recordings and—if available–results of imaging studies could be analyzed in more detail to shed light on the spectrum of this disease. Furthermore, it is of high interest to know the long-term consequences of patients who have suffered from COVID-19. The Chinese collegues could be pioneers in assessing these questions by carrying out follow-up investigations regarding pulmonary, cardiac, endocrinologic, neurologic, psychiatric, and immunologic impairments of post-COVID-19 patients (27).

## Data Availability Statement

The original contributions presented in the study are included in the article/supplementary materials, further inquiries can be directed to the corresponding author/s.

## Author Contributions

All authors listed have made a substantial, direct and intellectual contribution to the work, and approved it for publication.

## Conflict of Interest

The authors declare that the research was conducted in the absence of any commercial or financial relationships that could be construed as a potential conflict of interest.
